# PRDX3 Promotes Lymph Node Metastasis in Cervical Cancer by Activating NF-κB Signaling Pathway and Anoikis Resistance

**DOI:** 10.7150/ijms.118912

**Published:** 2025-08-22

**Authors:** Weijia Wen, Jiaying Li, Li Yuan, Yan Liao, Caixia Shao, Dingze Xu, Hongye Jiang, Yuandong Liao, Pan Liu, Chunyu Zhang, Shuzhong Yao, Wei Wang

**Affiliations:** 1 Department of Obstetrics and Gynecology, the First Affiliated Hospital, Sun Yat-sen University, Guangzhou, Guangdong, P.R. China.; 2 Guangdong Provincial Clinical Research Center for Obstetrical and Gynecological Diseases, Guangzhou, Guangdong, P.R. China.; 3 Department of Obstetrics and Gynecology, Dongguan Maternal and Child Health Care Hospital, Dongguan, Guangdong, P.R. China.

**Keywords:** Cervical cancer, Lymph node metastasis, PRDX3, NF-κB, Anoikis, ROS

## Abstract

Lymph node metastasis (LNM) confers significant treatment failure and adverse clinical outcomes in cervical cancer (CCa). However, large unknown lies in the mechanisms underlying LNM in CCa. In this study, we discovered that PRDX3 is elevated in CCa with LNM and associates with poor prognosis in CCa patients. Moreover, multivariate logistic analysis revealed that PRDX3 is an independent predictor of LNM in CCa. Functional investigations demonstrated that PRDX3 promotes invasion, lymphangiogenesis, and LNM of CCa. Mechanistically, PRDX3 activates NF-κB signaling pathway and upregulates the downstream pro-metastatic molecules VEGF-C and MMP-9 in CCa cells. Furthermore, PRDX3 could inhibit the anoikis of detached CCa cells by reducing the ROS level and hence promote LNM in CCa. Importantly, genetic inhibition of PRDX3 potently slows LNM of CCa. These findings highlight a novel PRDX3-mediated mechanism of LNM in CCa and recognize PRDX3 as a promising predictive marker and target of clinical intervention for LNM in CCa.

## Introduction

Cervical cancer (CCa) is on the top list of cancers in women worldwide. Its mortality ranked the 4th among female malignancies, greatly impacting the survival and life quality of CCa patients^1^. In 2022, there were 150 700 new cases of CCa and 55 700 deaths due to CCa in China with an increasing morbidity and mortality rate^2^. Lymph node metastasis (LNM) is a major cause for treatment failure and adverse clinical outcomes of CCa patients. The 5-year survival rate of CCa patients without LNM reaches 84.4%, while that of patients with LNM positive dramatically declines to 22.4%^3^. Nevertheless, there is still no valid measure to prevent or control LNM in CCa. Thus, it is imperative to dig into the exact molecular mechanisms of LNM in CCa so as to discover an effective therapeutic target.

Reactive oxygen species (ROS) are produced as significant byproducts of oxygen metabolism^4^. ROS has been gaining much attention for its context-dependent roles in tumor genesis and progression. Redundant ROS can result in cell cycle arrest and cell aging, exerting an anti-tumorigenic function. While on the other hand, the delicate manipulation of ROS balance facilitates cancer development by helping tumor cells escape from apoptosis and prolong survival^5^. Also, the dichotomized roles of ROS vary in different cancer stages. At early stages of cancer, ROS contributes to oxidation and mutations of specific tumorigenic oncogenes, resulting in tumor initiation^6^, while the accumulation of excess intracellular ROS can trigger apoptosis as cancer develops^7^. To adapt to oxidative stress, cancer cells evolve an ROS scavenging system to remove the over-accumulation of ROS^8^. Peroxiredoxins (PRDXs) are a family of thiol peroxidases that scavenge peroxides^9^. PRDXs can eliminate peroxide through catalyzing the oxidation of cysteine to sulfenic acid^10^. Distributed in different organelles, there are six isoforms of PRDX in mammals, of which PRDX3 is mainly in charge of the mitochondria^11^. A homeostasis of cellular ROS level mediated by PRDXs is required for cancer growth^4^. However, large unknown lies in how the ROS homeostasis is remodeled during the LNM in CCa.

In our previous research, we discovered that PRDX3 is significantly upregulated in CCa tissues with LNM^12^. Studies have showed that PRDX3 could eliminate 90% of cellular hydrogen peroxide^13^,^14^, protecting cells from oxidative damage and apoptosis^15^,^16^. In gastric cancer, upregulation of PRDX3 induced by anti-silencing function protein 1 homolog B (ASF1B) could stimulate tumor cells to proliferation and metastasize^17^. In glioblastoma, suppression of PRDX3 by silencing upstream prohibitin (PHB) could slow down tumor growth and improves radiotherapeutic efficacy^18^. Moreover, PRDX3 was found to take part in the formation of cervical intraepithelial neoplasia (CIN), where PRDX3 helped HPV persistent infection transform into CIN, and the PRDX3 expression level increased along as the grade of CIN progressed^19^,^20^. However, the biological function and mechanism of PRDX3 in LNM of CCa remains a mystery and needs to be unveiled.

Here in this study, for the first time we identified PRDX3 as a poor prognosis factor for CCa and independent predictor of LNM. PRDX3 was highly expressed in CCa with LNM relative to CCa without LNM. Functional experiments revealed the promoting effects of PRDX3 on the migration, invasion, lymphangiogenesis, and LNM in CCa. Mechanistically, PRDX3 exerted its promoting functions via enhancing the resistance to anoikis by inhibiting mitochondrial ROS accumulation and activating NF-κB signaling pathway in CCa cells. Targeting PRDX3 by genetic knockdown potently inhibited LNM in CCa, highlighting PRDX3 as a potential target of clinical intervention for LNM in CCa.

## Materials and Methods

### Human specimens

For PRDX3 mRNA level detection, we included 55 fresh CCa tissues, of which 13 were LNM positive and 42 were LNM negative, and 19 normal cervix tissues, which were acquired between April, 2023 and April, 2024 at the First Affiliated Hospital of Sun Yat-sen University. For immunohistochemistry, 280 cervical cancer tissues sections (52 with LNM positive and 228 were LNM negative) between 2011 and 2023 were obtained from the Department of Pathology. Tumor tissues were harvested in radical hysterectomy operations of CCa patients, who all had not received previous anti-tumor treatment. Normal cervix tissues were from patients who underwent hysterectomy for benign gynecological diseases like uterine leiomyoma and adenomyosis, but without cervical lesions. Human research was approved by the Ethical Review Committee of the First Affiliated Hospital of Sun Yat-sen University (Approval number: 2023-008; Date: March 17th, 2023) and conducted in compliance with the Declaration of Helsinki. All enrolled patients provided written informed consent.

### Cell lines and culture conditions

CCa cell lines, including MS751, HeLa, HeLa229, SiHa, and the human normal cervix epithelial cell line H8 were all purchased from the American Type Culture Collection (ATCC). DMEM (Gibco, USA) was used to culture the above cells, supplemented with 10% fetal bovine serum (FBS) (Gibco, USA), 1% non-essential amino acid (NEAA) (Corning, NY, USA), and 1% penicillin and streptomycin (NCMBio, Suzhou, China). Human lymphatic endothelial cells (HLECs) were purchased from ScienCell (USA) and cultured in the ECM (ScienCell, USA). All cells were maintained in an thermostatic incubator under 37°C with 5% CO2. In 2023, all of the involved cell lines were subjected to STR genotyping for identification and to mycoplasma detection. To achieve a detached condition, tumor cells were cultured on 1% agar-treated plate for 48h.

### Total RNA extraction and qRT-PCR

Total RNAs of tissues were extracted via a Trizol-chloroform-based protocol. Total RNA extraction from cells was achieved with an RNA-Quick Purification Kit (ESScience, Shanghai, China). After quantification with a Nanodrop2000 (ThermoFisher, USA), an equal amount of RNA from each sample was reverse transcribed into cDNA using the Evo M-MLV RT-PCR kit (Accurate Biology, Changsha, China). The cDNAs were subsequently applied for qRT-PCR analysis with SYBR Green Premix Pro Taq HS qPCR kit (Accurate Biology, Inc.) on a Step One Plus Real-Time PCR System (ThermoFisher, USA). All the involved primers were displayed in Table S1. GAPDH was employed as an internal control. The 2(-ΔΔCt) method was applied for determining relative expression level.

### Immunohistochemistry (IHC)

5-μm paraffin-embedded tissue sections were subjected to IHC staining. In brief, after deparaffinization, rehydration, and sealing with goat serum, slides were incubated with a primary antibody overnight at 4°C, followed by incubation in a corresponding HRP-conjugated secondary antibody for 1h. Afterwards, staining was detected by DAB assay. After reverse dehydration and mounting, staining was scanned and analyzed. The antibodies involved in this study were displayed in Table S2. The IHC staining score, ranging from 0 to 6, was evaluated by two experienced pathologists as the sum of the area points (0% of cells stained gets 0 points, 0%-30% stained gets 1 point, 30%-60% stained gets 2 points, 60%-100% stained gets 3 points) and the intensity points (no staining gets 0 points, weak staining gets 1 point, moderate staining gets 2 points, strong staining gets 3 points). According to the results, 5 was set as the cutoff score to divide into low expression group and high expression group.

### Western-blot

A protease inhibitor-containing RIPA lysis buffer (CWBIO, Beijing, China) was used to extract the total proteins from cells. After quantification with a BCA kit (Thermo Fisher Scientific, Inc.), total proteins were separated by SDS-poly-acrylamide gels and transferred electrophoretically to the PVDF membrane (Merck Millipore, USA), which was incubated in sequence with 5% BSA (Beyotime, Shanghai, China), primary antibody and secondary antibody and detected by ECL Western Blotting Substrate (Merck Millipore, USA). Image J software was used to quantify the stripes.

### Transwell assay and HLEC tube formation assay

For transwell invasion assay, each upper chamber was precoated with Matrigel (Corning, USA) and seeded 5×104 starved tumor cells. The upper chamber contained serum-free medium and the lower chamber was dipped in complete medium with 15% FBS. After being cultured under 37°C for 48h, migrated cells on the lower chamber were counted under a light microscope after fixation with paraformaldehyde and staining with crystal violet. The procedures of transwell migration were the same as above expect that the upper chambers were not coated with Matrigel. For HLECs transwell assay, 3×104 HLECs were seeded and incubated for 24h before detection. For tube formation assay, HLECs were seeded onto the Matrigel-precoated 96-well plate at the density of 1×104 cells/well, cultured with ECM (Gibco, USA) containing tumor cells culture supernatant under 37°C for 4h-6h. The total length of formed tube structures was measured under a light microscope. Image J was used to process the data.

### Xenograft nude mouse LNM model

All the animal experiments were approved by the Animal Ethics Committee of Sun Yat-sen University (Approval number: SYSU-IACUC-2022-001358; Date: September 16th, 2022). All the xenograft tumors in this study didn't exceed the maximal tumor size permitted by the ethics committee, which is no diameter shall greater than 20 mm nor weigh greater than 10% of its body weight. Xenograft LNM model was established on female BALB/c nude mice. About an amount of 6×106 SiHa cells were injected into the mice foot-pads. BAY-11-7082 (Aladdin, Shanghai, China) or placebo was administered by intra peritoneal injection twice a week at the dose of 5mg/kg since the 39th day after tumor cell inoculation. On the 60th day, the mice were sacrificed and removed of their popliteal lymph nodes and primary foot-pad tumors, whose calibre was measured for volume evaluation as: volume (mm3) = 0.52 × (length [mm]) × (width [mm])2. The harvested specimens were fixed with formalin and made into paraffin-embedded sections for HE staining and IHC.

### PRDX3 expression manipulation

To knockdown PRDX3 expression, two different siRNAs targeting PRDX3 were synthesized by GenePharma (Suzhou, China). Lipofectamine RNAiMAX (Invitrogen, USA) was used to transfect tumor cells with above siRNAs. These two siRNAs were provided in Table S3. The shPRDX3 plasmid and PRDX3 overexpression plasmid were synthesized by Vigene Biosciences (Shandong, China) and co-transfected with psPAX2 and pMD2.G into 293T cells using X-tremeGENE HP DNA transfection reagent (Roche, Switzerland). Lentivirus-packaged shPRDX3 and PRDX3 overexpression plasmids were harvested 48h and 72h after transfection and infected tumor cells with complete medium containing 10 μg/ml Polybrene (Beyotime, Shanghai, China). The stably and successfully manipulated cells were singled out for 5 days under the condition of puromycin (2 μg/ml, Sigma-Aldrich, USA).

### ROS detection and apoptosis detection

For intracellular ROS level examination, a Reactive Oxygen Species Assay Kit (Beyotime, Shanghai, China) was applied according to the manufacturer's instructions. In brief, serum-free culture medium diluted DCFH-DA was used to suspend tumor cells and incubate under 37°C and 5% CO2 for 20 minutes. After being washed with serum-free medium, then tumor cells were subjected to flow cytometry analysis. For mitochondrial ROS detection, Mitochondrial Superoxide Assay Kit with MitoSOX Red (Invitrogen, USA) was applied. In brief, MitoSox Red was diluted into 5μM with HBSS (Invitrogen, USA) and then incubated cells for 10 minutes from light before flow cytometry analysis. For apoptosis detection, an Annexin V-FITC/PI Apoptosis Detection Kit (Vazyme, Nanjing, China) was utilized according to the manufacturer's protocols. In short, suspended and washed cells were incubated with 5μl PI Staining Solution and 5μl Annexin V-FITC in 100μl Binding Buffer for 10 minutes at room temperature from light and then immediately submitted to flow cytometry analysis on CytoFLEX machine (Beckman Coulter, USA).

### Statistical analysis

SPSS 13.0 (SPSS Inc., Chicago, USA) and GraphPad Prism 9.5 software were used for statistical analysis and plotting. Student's t-test was employed for differentiating the averages of two separate groups. Pearson χ2 test or Fisher's exact test were the method of evaluating relationships between clinicopathological characteristics and PRDX3 expression. The Kaplan-Meier method was employed in overall survival (OS) and recurrence-free survival (RFS) analyses. Log-rank test was used to compare the curves of different groups. Spearman rank correlation analysis was utilized for analysis of correlations between measured variables. Multivariate logistic regression was employed to recognize independent predictors for LNM in CCa. Receiver operating characteristic (ROC) analysis was for determination of the discriminatory power of specific indicators. Cox proportional hazard model was employed to discriminate independent predictive factors for prognosis in CCa patients. P < 0.05 was set as the cutoff to determine statistical significance.

## Results

### PRDX3 is correlated with LNM in CCa

**Figure 1:**
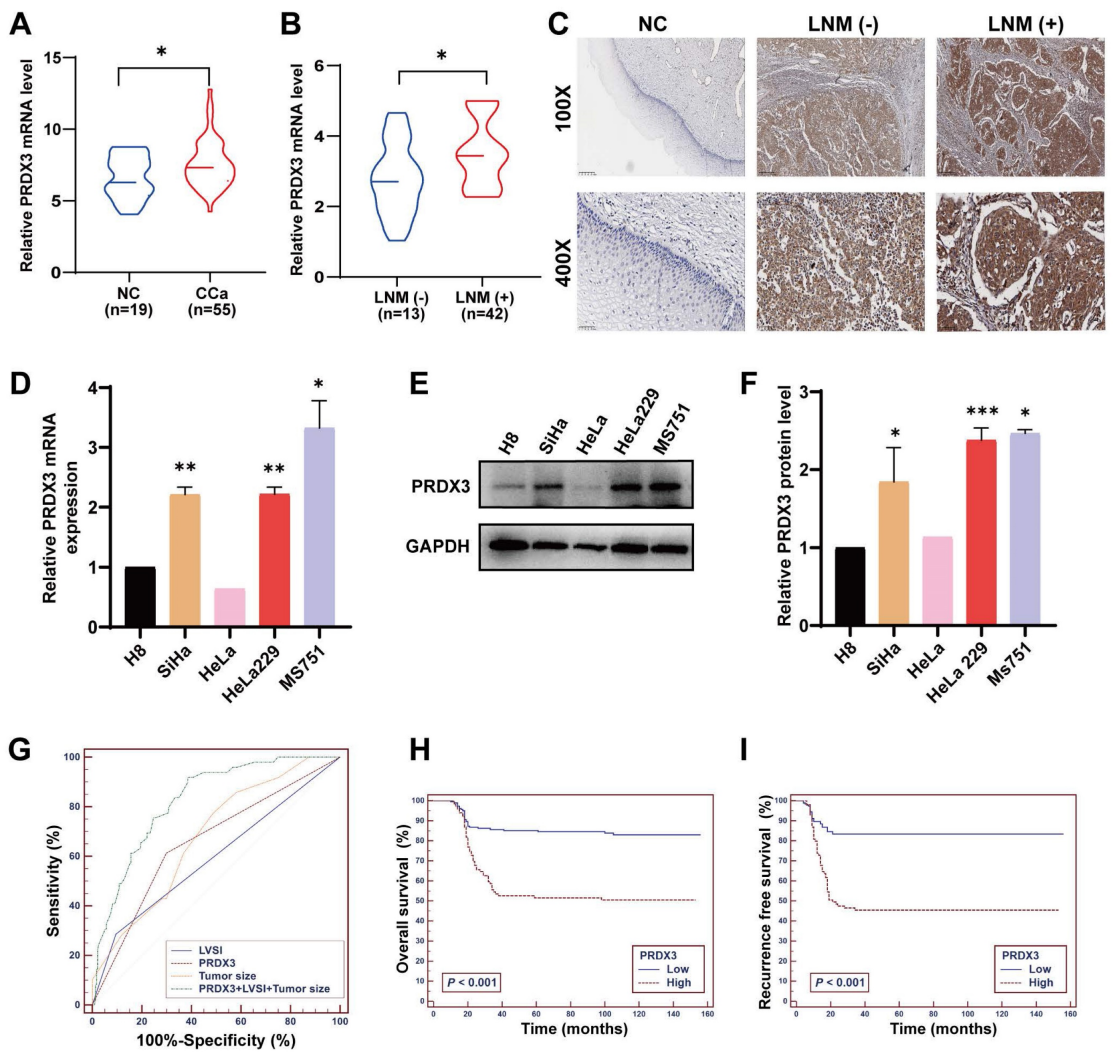
** PRDX3 is associated with lymph node metastasis and poor prognosis in CCa.** (A) The relative PRDX3 mRNA level is higher in CCa tissues (n = 55) than in normal cervix tissues (n = 19). (B) The relative PRDX3 mRNA level is higher in CCa tissues with LNM (+) (n = 13) than those with LNM (-) (n = 42). (C) Representative images of IHC staining of PRDX3 in normal cervix tissues, CCa tissues with LNM (-) and CCa tissues with LNM (+). (D) Relative PRDX3 mRNA expression levels in CCa cell lines and normal cervix cell line H8. (E)-(F) Relative PRDX3 protein level in CCa cell lines and normal cervix cell line H8. (G) ROC curves of PRDX3, LVSI and tumor size as predictive markers for LNM in CCa. (H)-(I) Kaplan-Meier survival curve of OS and RFS of CCa patients grouped by PRDX3 IHC scores. Error bars represent the mean ± S.D. of three independent experiments. * P < 0.05, ** P < 0.01, *** P < 0.001.

To explore whether PRDX3 plays an important role in LNM of CCa, we investigated the level of PRDX3 protein and mRNA expression in CCa tissues. Results showed that the mRNA and protein level of PRDX3 in CCa tissues with LNM were both significantly higher than those in CCa tissues without LNM and normal cervix (Fig. 1A-C). The representative PRDX3 protein levels were displayed by IHC staining in Fig. 1C. We then detected the PRDX3 expression in normal cervical epithelial cell line H8 and several CCa cell lines. The expression levels of PRDX3 mRNA and protein were significantly higher in CCa cell lines than those in H8 (Fig. 1D-F). Moreover, the mRNA and protein expression levels of PRDX3 in MS751, a CCa cell line from metastatic lymph nodes, were detected higher than those in HeLa and SiHa, which were derived from primary locus (Fig. 1D-F). These results suggested that PRDX3 may promote lymph node metastasis in CCa.

Next, we determined to evaluate the correlations between PRDX3 expression and clinical characteristics of CCa patients. We performed IHC staining of PRDX3 and scored its expression on 280 paraffin-embedded CCa sections, and its correlation to clinicopathological factors was examined. The results indicated that PRDX3 was significantly correlated with FIGO stage (P = 0.001), tumor size (P < 0.001), LNM (P < 0.001), and lymphovascular space invasion (LVSI) (P = 0.042) in CCa (Table 1).

To further investigate whether PRDX3 is an independent risk factor for LNM in CCa, we conducted multivariate logistic regression analysis and found that all of LVSI, tumor size and PRDX3 expression possess independent predictive power for LNM in CCa (Table 2). Furthermore, we constructed ROC curves of these three markers, assessing the discriminatory power of LVSI, tumor size, and PRDX3 expression to predict LNM in CCa (Fig. 1G). Subsequently, a logistic regression model was carried out to further evaluate the feasibility of the combination of LVSI, tumor size, and PRDX3 expression to predict LNM in CCa. The positive predictive value, negative predictive value, sensitivity, specificity, and area under the ROC curve of LVSI, tumor size and PRDX3 separately, as well as their combination are shown in Table 3. When LVSI, tumor size, and PRDX3 were combined, the predictive power for LNM in CCa was strong (AUC = 0.826) (Table 3).

### Elevated PRDX3 confers higher postoperative relapse and decreased survival in CCa patients

Of the 280 enrolled CCa patients, 201 (71.79 %) were still alive as of current statistics, 79 (28.21 %) had deceased from CCa, and a total of 84 (30 %) underwent disease recurrence. To evaluate the potential of PRDX3 being a prognostic factor in CCa, we constructed a multivariate Cox proportional hazard model, where age, tumor size, LNM, LVSI and PRDX3 were identified as independent prognostic factors for OS in CCa patients, and tumor size, LNM, LVSI, as well as PRDX3 were confirmed as independent prognostic factors for RFS (Table 4). Kaplan-Meier survival curve analysis revealed that CCa patients with high PRDX3 expression suffered from markedly declined OS and RFS (Fig. 1H-I).

### PRDX3 promotes *in vitro* invasion ability and *in vivo* LNM of CCa cells

**Figure 2:**
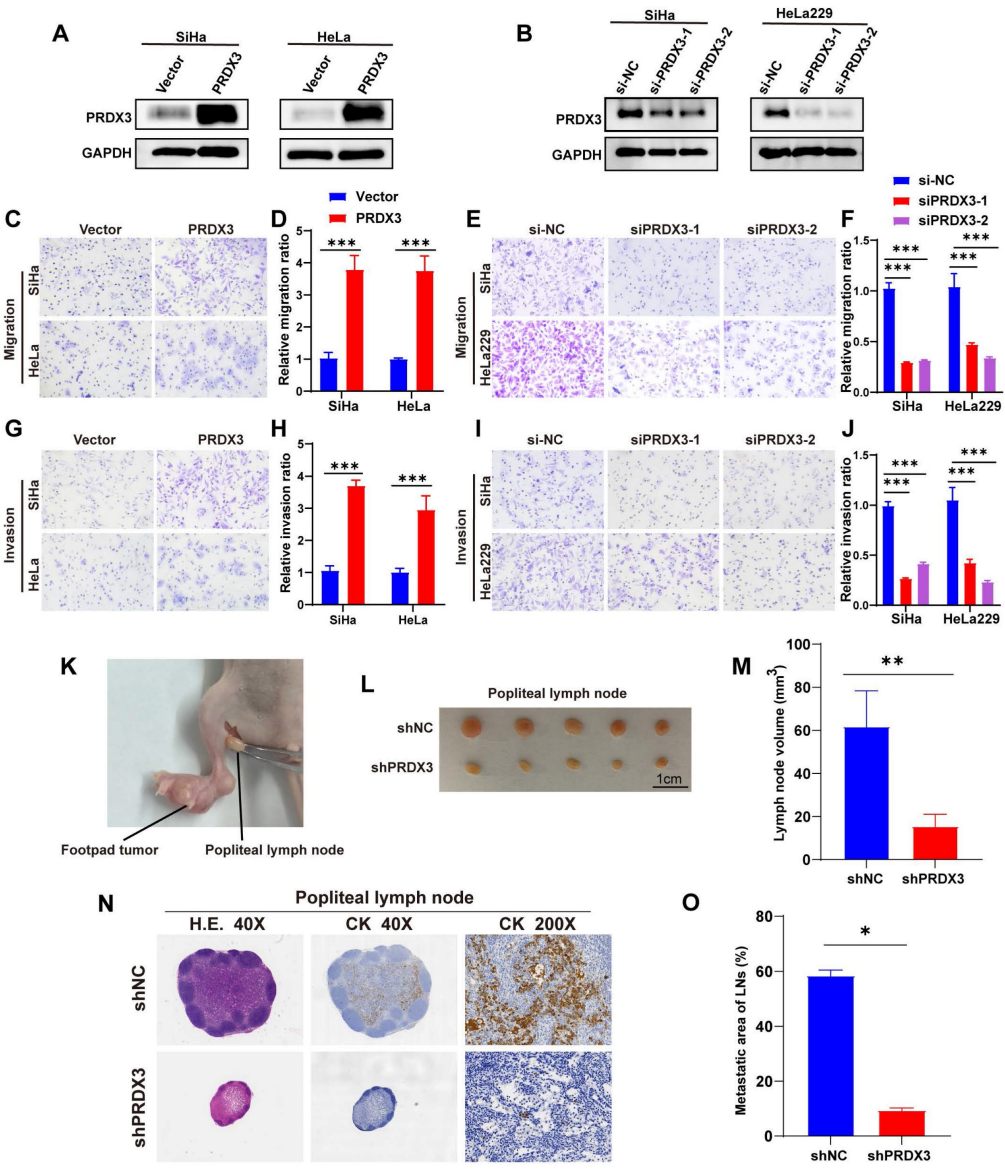
** PRDX3 promotes invasion ability of CCa cells *in vitro* and lymph node metastasis of CCa cells *in vivo*.** (A) Overexpressing PRDX3 significantly elevated the protein level of PRDX3. (B) Silencing PRDX3 using siRNAs significantly reduced the protein level of PRDX3. These experiments were repeated three times independently. (C)-(D) Overexpressing PRDX3 promoted the migration capacity of CCa cells. (E)-(F) Silencing PRDX3 expression inhibited the migration capacity of CCa cells. (G)-(H) Overexpressing PRDX3 promoted the invasion capacity of CCa cells. (I)-(J) Silencing PRDX3 expression inhibited the invasion capacity of CCa cells. (K) A nude mouse LNM model was applied. (L) Representative images of popliteal lymph nodes in the shNC and shPRDX3 groups. (M) Histogram analysis of popliteal lymph nodes volumes (n = 10 mice per group). (N) Representative H.E and IHC images of pan-cytokeratin in popliteal lymph nodes of the shNC and shPRDX3 groups. (O) Histogram analysis of the metastatic area of LNM in nude mice model (n = 10 mice per group). Error bars represent the mean ± S.D. of three independent experiments. * P < 0.05, ** P < 0.01, *** P < 0.001.

To confirm the cancer-promoting biological function of PRDX3 in LNM of CCa, we conducted loss-of-function and gain-of-function assays in CCa cell lines. According to their primary expression level of PRDX3 as shown in Fig 1D-1F, we chose SiHa and HeLa for PRDX3 overexpression, while SiHa and HeLa229 for PRDX3 knockdown. The efficiencies of PRDX3 overexpression and knockdown were verified (Fig. 2A-B). Transwell assay was utilized for migration and invasion ability detection. Our data showed that PRDX3 overexpression significantly promoted migration and invasion of SiHa and HeLa (Fig. 2C-D, 2G-H), while PRDX3 knockdown significantly inhibited migration and invasion ability of SiHa and HeLa229 (Fig. 2E-F, 2I-J), suggesting that PRDX3 could facilitate metastatic ability of CCa cells *in vitro*.

Next, we apply a LNM model on nude mice to further evaluate the effect of PRDX3 on LNM in CCa (Fig. 2K). We inoculated shPRDX3 SiHa cells and the corresponding normal control cells into the foot-pads of nude mice, and collected popliteal lymph nodes for assessment at an appropriate timepoint. As shown in Fig. 2L-O, PRDX3 knockdown markedly inhibited the LNM capacity of CCa cells. The volumes of popliteal lymph nodes were decreased prominently on PRDX3 knockdown (Fig. 2L-M). Pan-cytokeratin staining demonstrated that as compared to the control group, the LNM areas were also decreased in the PRDX3 knockdown group (Fig. 2N-O). Together, our findings supported that PRDX3 could promote *in vivo* LNM of CCa cells.

### PRDX3 stimulates lymphangiogenesis of CCa *in vitro* and *in vivo*

**Figure 3:**
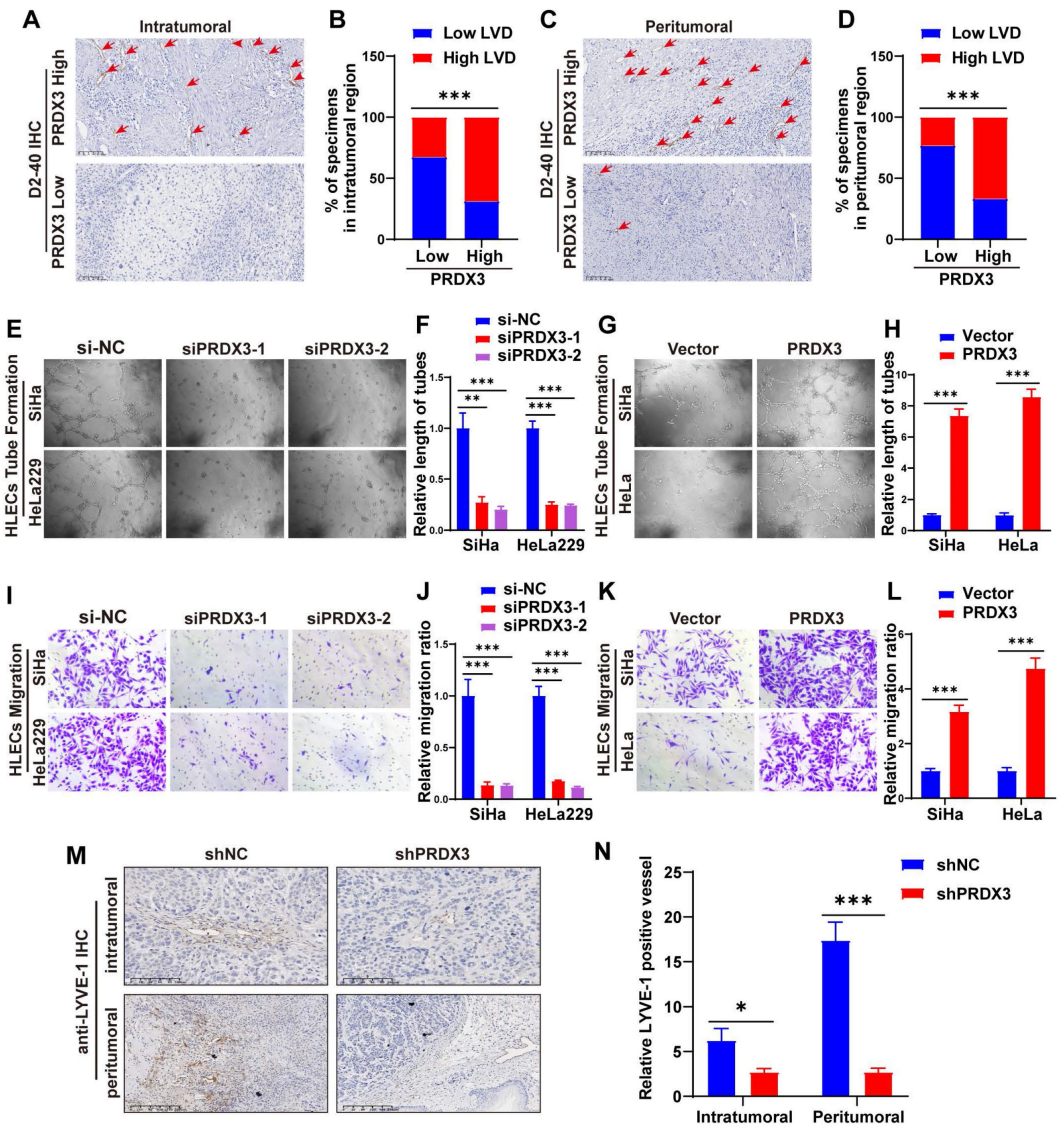
** PRDX3 stimulates lymphangiogenesis of CCa *in vitro* and *in vivo*.** (A)-(D) CCa tissues highly expressing PRDX3 manifest a higher lymphatic vascular density (LVD) in both the intratumoral region and peritumoral region. (E)-(H) The effect of PRDX3 on the tube formation of HLECs (×200). (I)-(J) The culture medium of CCa cells with PRDX3 knockdown significantly inhibited the migration capacity of HLECs. (K)-(L) The culture medium of CCa cells overexpressing PRDX3 significantly augmented the migration capacity of HLECs. (M) Representative IHC staining of LYVE-1 in primary tumors in nude mice model (×200). (N) Histogram analysis of LYVE-1 positive vessels in the nude mice model (n = 10 mice per group). Error bars represent the mean ± S.D. of three independent experiments. ** P < 0.01, *** P < 0.001.

Lymphangiogenesis is a pivotal process during LNM of cancer^8^,^9^. We performed IHC staining of D2-40, a marker for lymphatic endothelial cells, on CCa tissue slides and found that the lymphatic vessel density (LVD) in both the intratumoral and the peritumoral regions were significantly higher in PRDX3 high expression group, suggesting that PRDX3 may be critical in lymphangiogenesis in CCa (Fig. 3A-D). Thus, we investigated the effect of PRDX3 on lymphangiogenesis of CCa *in vitro* by tube formation assays. Our data indicated that the conditioned medium from the PRDX3-overexpression CCa cells strongly promoted tube formation of HLECs, whereas PRDX3 knockdown impaired the ability of cervical cancer cells to induce HLEC tube formation (Fig. 3E-H). Additionally, transwell assays revealed that overexpressing PRDX3 in CCa cells markedly promoted the migration capacity of HLECs, while depleting PRDX3 in CCa cells generated the opposite effect (Fig. 3I-L). *In vivo* animal models also demonstrated that the intra-tumoral and peri-tumoral lymphatic vessels in the primary tumors, as indicated by IHC staining against a lymphatic marker, lymphatic vessel endothelial hyaluronan receptor 1 (LYVE-1), significantly declined in the mice bearing PRDX3 knockdown CCa cells (Fig. 3M-N). These findings collectively indicated that PRDX3 stimulates lymphangiogenesis of CCa *in vitro* and *in vivo*.

### PRDX3 promotes CCa invasion, lymphangiogenesis and LNM via activating NF-κB signaling pathway

**Figure 4:**
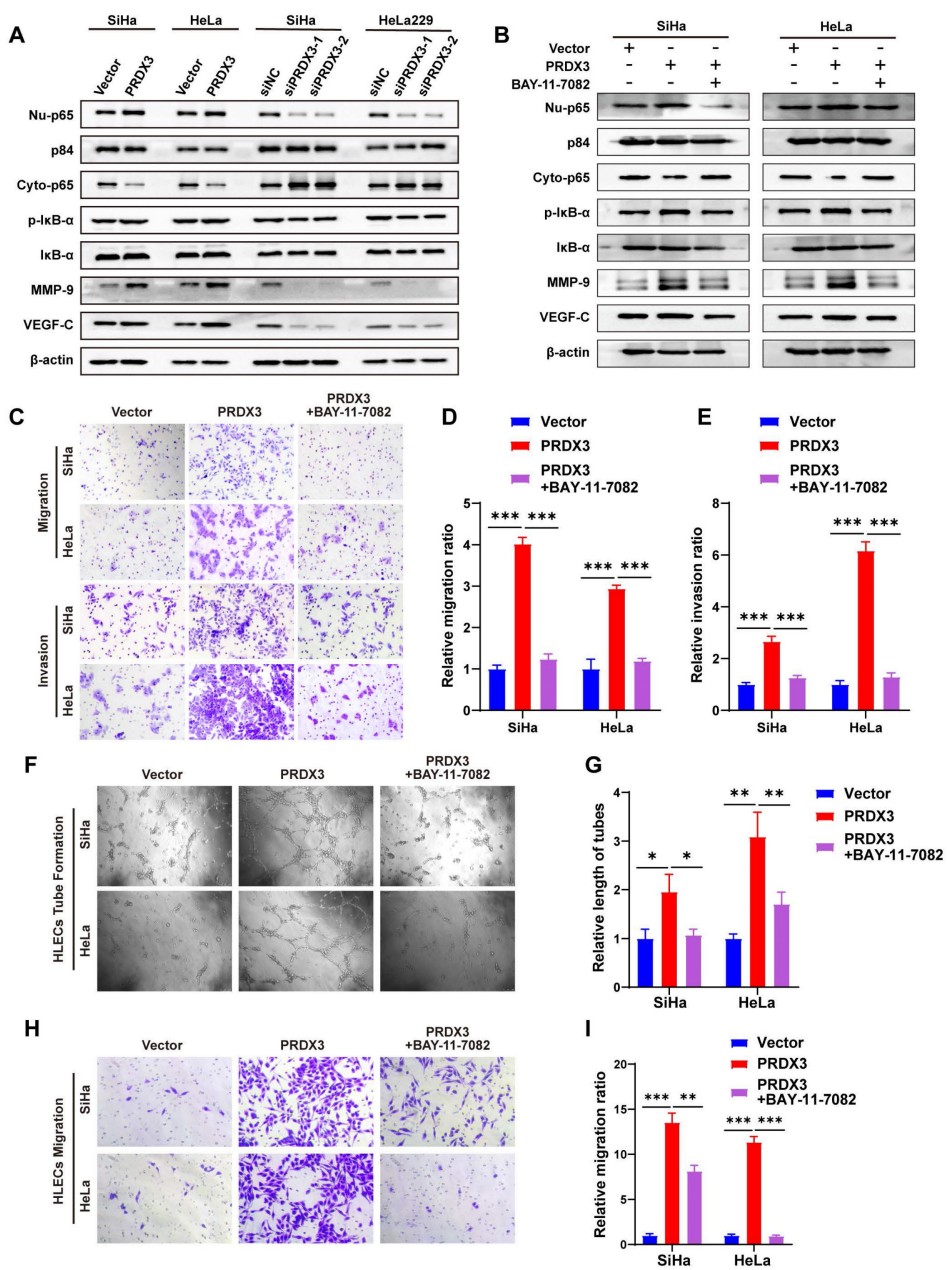
** PRDX3 promotes CCa invasion, lymphangiogenesis of CCa cells *in vitro* via activating NF-κB signaling pathway.** (A) Western Blot analysis of the effect of PRDX3 on the expression of NF-κB signaling pathway molecules and downstream metastasis-related targets MMP-9 ad VEGF-C. The nuclear protein p84 was used as the nuclear protein markers. Nu-p65, Nuclear p65; Cyto-p65, cytoplasmic p65. (B) Western Blot analysis of NF-κB signaling pathway molecules and downstream metastasis-related targets MMP-9 ad VEGF-C after treating the indicated cells with a NF-κB signaling inhibitor BAY-11-7082. (C)-(E) NF-κB signaling inhibitor BAY-11-7082 attenuated the stimulatory effects of PRDX3 on the migration and invasion abilities of CCa cells. (F)-(G) NF-κB signaling inhibitor BAY-11-7082 attenuated the stimulatory effect of PRDX3 on the tube formation of HLECs (×200). (H)-(I) NF-κB signaling inhibitor BAY-11-7082 attenuated the stimulatory effect of PRDX3 on the migration ability of HLECs. Error bars represent the mean ± S.D. of three independent experiments. * P < 0.05, ** P < 0.01, *** P < 0.001.

Our previous study discovered that NF-κB signaling pathway activation by fatty acid binding protein 5 (FABP5) promoted CCa invasion and lymphangiogenesis^21^. We wondered whether PRDX3 also exerts its pro-metastatic functions via NF-κB signaling pathway. We found that augmenting the expression of PRDX3 could increase, while silencing PRDX3 could decrease the protein level of Nu-p65 and p-IκB-α. A few of downstream metastasis-related target molecules in NF-κB signaling, including vascular endothelial growth factor C (VEGF-C) and matrix metallopeptidase-9 (MMP-9), were also found increased after overexpressing PRDX3, but reduced after PRDX3 silencing (Fig. 4A). These findings indicated that PRDX3 could activate NF-κB signaling pathway in CCa cells.

**Figure 5:**
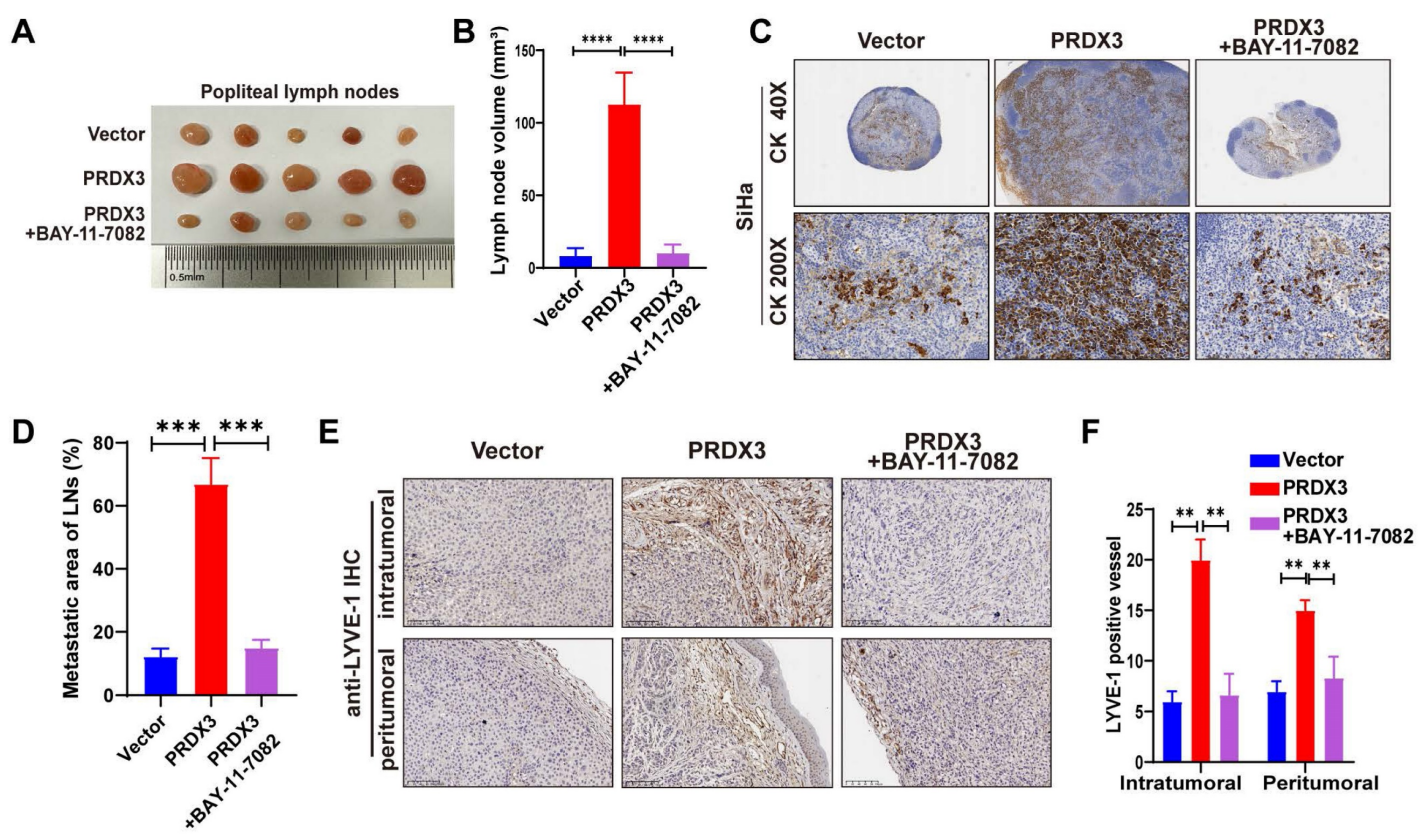
** PRDX3 promotes LNM and lymphangiogenesis *in vivo* via an NF-κB signaling pathway-dependent way.** (A)-(B) NF-κB signaling inhibitor BAY-11-7082 attenuated the stimulatory effect of PRDX3 on the volumes of popliteal lymph nodes in nude mice model (n = 10 mice per group). (C)-(D) NF-κB signaling inhibitor BAY-11-7082 attenuated the stimulatory effect of PRDX3 on the metastatic areas of LNM in nude mice model (n = 10 mice per group). (E)-(F) NF-κB signaling inhibitor BAY-11-7082 attenuated the stimulatory effect of PRDX3 on lymphangiogenesis in the primary tumors of nude mice (×200). Error bars represent the mean ± S.D. of three independent experiments. ** P < 0.05, *** P < 0.01, **** P < 0.001.

Further, we probed into the significance of NF-κB signaling pathway in the pro-metastatic function of PRDX3 in CCa cells by means of a NF-κB signaling inhibitor BAY-11-7082. Treatment with BAY-11-7082 significantly reduced the protein level of Nu-p65, p-IκB-α, MMP-9 and VEGF-C in HeLa and SiHa cells (Fig. 4B). The results of transwell assay indicated that repressing NF-κB signaling by BAY-11-7082 reversed the promoting effect of overexpressing PRDX3 expression on the migration and invasion capacity of CCa cells (Fig. 4C-E). Tube formation assay demonstrated that overexpressing PRDX3 significantly promoted the length of formed lymphatic tubes, while the additional treatment with BAY-11-7082 reversed this trend in CCa cells (Fig. 4F-G). HLECs transwell assay showed that BAY-11-7082 weakened the promoting effect of PRDX3 on the migration capacity of HLECs (Fig. 4H-I). Additionally, BAY-11-7082 impaired the enhancement of lymph node volumes and metastatic areas of lymph nodes brought by forced PRDX3 expression in nude mice bearing CCa cells (Fig. 5A-D). Same phenomena were observed in the promoting function of PRDX3 on the number of lymphatic vessels as assessed by LYVE-1 staining *in vivo* in CCa (Fig. 5E-F). Taken these results together, it was indicated that PRDX3 exerted its pro-metastatic function in CCa cells in a NF-κB-signaling-dependent manner.

### PRDX3 enhances resistance to anoikis by reducing ROS in detached CCa cells

**Figure 6:**
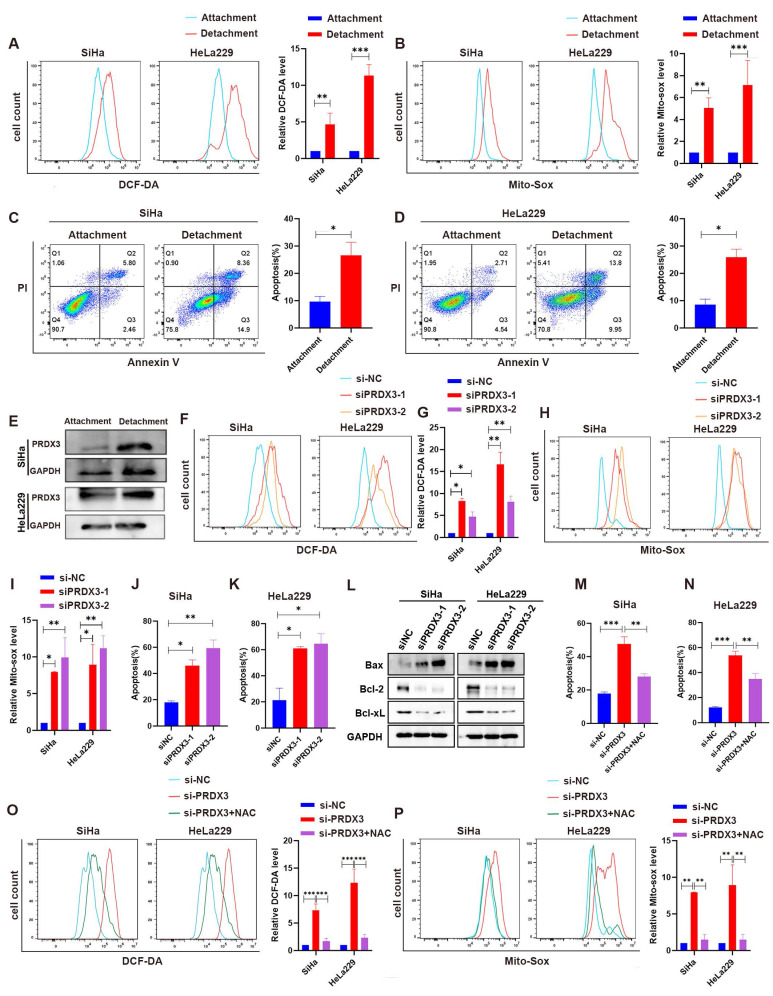
** PRDX3 mitigates anoikis by reducing ROS level in detached CCa cells.** (A) The intracellular ROS level of CCa cells in the attached and detached states. (B) The mitochondrial ROS level of CCa cells in the attached and detached states. (C)-(D) Flow cytometry analysis for apoptosis detection of CCa cells in the attached and detached states. (E) The PRDX3 protein level of CCa cells in the attached and detached states. (F)-(G) The effect of PRDX3 on the intracellular ROS level of detached CCa cells. (H)-(I) The effect of PRDX3 on the mitochondrial ROS level of detached CCa cells. (J)-(L) Silencing PRDX3 in detached CCa cells significantly elevated the anoikis proportions. (M)-(N) ROS-ridding antioxidant NAC attenuated the enhancing effect on the intracellular and mitochondrial ROS level in detached CCa cells brought by silencing PRDX3. (O)-(P) ROS-ridding antioxidant NAC attenuated the enhancing effect on the anoikis proportions in detached CCa cells brought by silencing PRDX3. Error bars represent the mean ± S.D. of three independent experiments. * P < 0.05, ** P < 0.01, *** P < 0.001.

Evidence have been found that acquaintance of resistance to anoikis could help tumor cell to metastasize^22^-^24^. As tumor cells detach from primary sites for metastasis, ROS is believed to be enriched, may accelerate tumor cell anoikis and promote metastasis^25^,^26^. We wondered whether highly-expressed PRDX3, as a significant regulator for ROS level, could help resist to anoikis during metastasis in CCa cells. For this purpose, we cultured CCa cells under detached conditions and inspected the ROS level in CCa cells. We found that the intracellular ROS level and mitochondrial ROS lever were both significantly higher in SiHa and HeLa229 cells in the detachment status than those in the attachment status (Fig. 6A-B). Meanwhile, the apoptosis proportion were also higher when SiHa and HeLa229 cells were in the detachment status (Fig. 6C-D). Interestingly, the expression level of PRDX3 was also robustly higher in CCa cells of detached condition than that in attached condition (Fig. 6E). We then further silenced PRDX3 expression in detached CCa cells and found that the intracellular ROS level, the mitochondrial ROS level and the apoptosis proportion were all elevated after PRDX3 knockdown (Fig. 6F-L). These results suggested that PRDX3 could elevate ROS level and enhance resistance to anoikis in CCa cells.

To explore whether PRDX3 promotes resistance to anoikis in CCa cells in an ROS-dependent manner, we employed N-Acetyl-L-cysteine (NAC), an ROS-ridding antioxidant for rescue assays. Supplement with NAC remarkably reversed the intracellular and mitochondrial ROS level and the apoptosis proportion in SiHa and HeLa229 cells (Fig. 6M-P). Collectively, these results confirmed that PRDX3 contributed to the acquisition of anoikis resistance in detached CCa cells by reducing ROS level.

## Discussion

**Figure 7:**
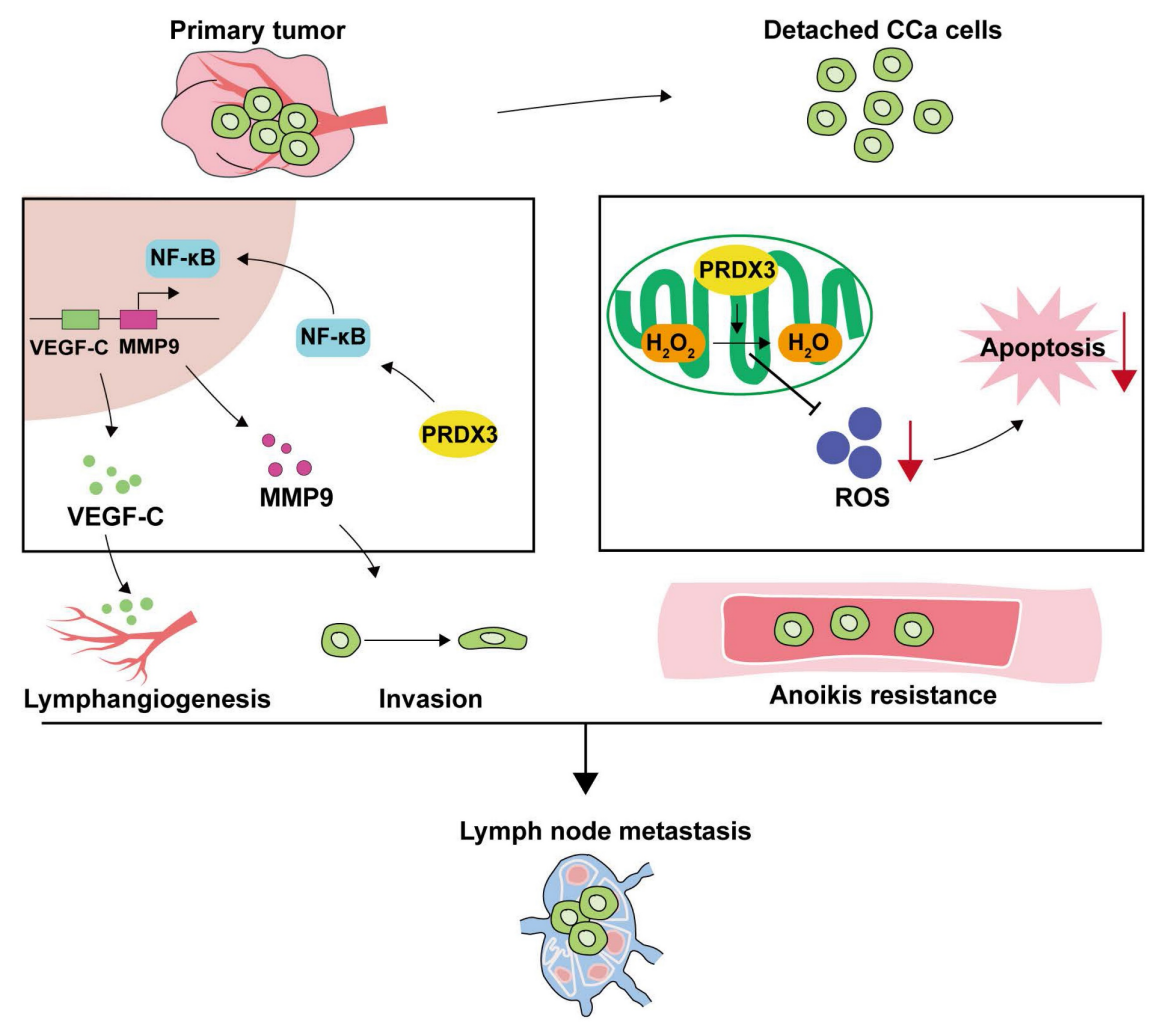
** Schematic illustration showing the proposed mechanisms by which PRDX3 promotes lymph node metastasis in CCa.** In primary tumors, highly-expressed PRDX3 activates the NF-κB signaling pathway where the downstream molecules MMP-9 and VEGF-C promote the invasion ability and lymphangiogenesis in CCa. In detached CCa cells, elevated PRDX3 hinders cell apoptosis by reducing ROS level. Together, these two pathways support the promotion effect of PRDX3 on LNM in CCa.

LNM is one of the most common clinicopathological features that confer poor prognosis in CCa patients, yet the existing therapeutic strategies yield unsatisfactory outcomes for these patients^27^. Therefore, a full-scale understanding of the underlying molecular mechanisms may offer insights into strategies for prevention and treatment in CCa patients with LNM. Herein, we discover the elevated PRDX3 in CCa tissues and cell lines with LNM. The aberrant expression of PRDX3 in CCa significantly predicts LNM and poor prognosis. Moreover, PRDX3 promotes primary tumor cell invasion, lymphangiogenesis and LNM in CCa via NF-κB signaling pathway. Furthermore, PRDX3 could resist anoikis of detached CCa cells by reducing the ROS level and promoted LNM in CCa (Fig. 7).

As a natural scavenger of peroxides, PRDX3 can protect cells from oxidative stress. Several researches have demonstrated that PRDX3 may take an important part in malignancies. In colorectal cancer, upregulation of PRDX3, induced by forkhead box M1 (FOXM1), could stimulate tumor cells to multiply and metastasize to distant locations by mitochondrial dysfunction^28^. In triple-negative breast cancer, PRDX3 regulates tumor metastasis via ERK signaling-mediated MMP-1 expression^29^. Nevertheless, the association between PRDX3 and LNM in CCa has not been investigated. In this study, we discover that PRDX3 is upregulated in CCa patients with LNM and correlates with poor prognosis. CCa cell lines derived from LNM sites also characterizes a higher mRNA and protein level of PRDX3. More importantly, multivariate logistic analysis reveals the independently predictive function of PRDX3 in CCa LNM. These findings indicate that PRDX3 may serve as an important facilitator in LNM of CCa.

Tumor cells must negotiate a series of steps to achieve metastases, each requiring specific functions, including detaching from primary site, migration and invasion, circulation, extravasation and seeding to colonize distant organs^30^. When tumor cells detach from primary environment and enter the circulation, anoikis occur along with a series of intracellular signaling pathway changes, including a higher level of ROS. Tumor cells need more antioxidants to maintain a redox balance to survive the anoikis^25^. In non-small cell lung cancer, suppression of PRDX3 induced by a ubiquitin specific peptidase-7 (USP7)-mediated manner could mitigate intracellular ROS and promote Osimertinib resistance^31^. In ovarian cancer, PRDX3 drives tumor progression by suppressing ROS accumulation and fostering ferroptosis resistance^32^. These findings supported that PRDX3 could urge tumor progression by reducing ROS. However, the role of PRDX3 in LNM of CCa is not yet fully understood. In our study, we silenced PRDX3 in detached CCa cells and detected an increase in intracellular ROS and mitochondrial ROS accompanied by a higher proportion of tumor cell apoptosis. While additional treatment with an antioxidant NAC could reverse the changes in ROS level and apoptosis proportion caused by silencing PRDX3 in detached CCa cells. These results demonstrate that PRDX3 could mitigate the ROS level in detached CCa cells to acquire the resistance to anoikis, which might facilitate LNM in CCa.

Another momentous finding from this study is that the pro-metastatic function of NF-κB pathway in CCa could be mediated by elevated PRDX3. It is reported that the excessive NF-κB pathway activation is correlated to LNM in breast cancer^33^,^34^, bladder cancer^35^ and cholangiocarcinoma^36^. Vital biological processes mediated by the downstream effector molecules in the NF-κB signaling pathway are essential for the cancer metastasis^37^-^39^. Our previous study showed that FABP5, also through NF-κB signaling pathway activation induced by excessive fatty acid, stimulated tumor cell epithelial-mesenchymal transition (EMT), invasion and lymphangiogenesis in CCa^21^. These findings provide potent evidence that NF-κB signaling pathway is pivotal in LNM of CCa. Here in this study, we noticed that NF-κB signaling pathway was activated when PRDX3 was artificially augmented in CCa cells. The pro-metastatic effector molecules like VEGF-C and MMP-9 in NF-κB signaling pathway were also upregulated by elevated PRDX3. In addition, rescue supplement of NF-κB pathway inhibitor BAY-11-7082 attenuated the facilitating effect of PRDX3 in CCa LNM. Our above findings verify that PRDX3 promotes CCa cells aggressiveness through activating the NF-κB signaling pathway, resulting in CCa LNM, which significantly expands the current knowledge of NF-κB pathway regulation in CCa.

## Conclusions

In summary, we have elucidated a new PRDX3-mediated mechanism of LNM in CCa, where PRDX3 could promote tumor cell invasion, lymphangiogenesis and LNM via NF-κB signaling pathway and scavenging ROS level to resist anoikis. Moreover, our study confirms the potential of PRDX3 being a predictive biomarker and therapeutic target for CCa patients with LNM.

## Supplementary Material

Supplementary tables.

## Figures and Tables

**Table 1:** Associations of PRDX3 expression levels with clinicopathological factors in early-stage cervical cancer.

	N	PRDX3		*P*
	280	Low (0-4)^a^ 181	High (5-6)^a^ 99	
Age (years)				0.962
≤ 35	57	37	20	
> 35	223	144	79	
FIGO stage				0.001
Ⅰa2	22	21	1	
Ⅰb1	133	91	42	
Ⅰb2	54	30	24	
Ⅱa1	39	27	12	
Ⅱa2	32	12	20	
Tumor size (cm)				< 0.001
≤ 4	194	139	55	
> 4	86	42	44	
LNM				< 0.001
Positive	52	19	33	
Negative	228	162	66	
LVSI				0.042
Positive	38	19	19	
Negative	242	162	80	
Differentiation				0.541
G1	18	11	7	
G2	98	59	39	
G3	164	111	53	
Stomal invasion				0.081
≤ 1/2	140	93	47	
> 1/2	140	88	52	
Parametrial invasion				0.597
Positive	17	12	5	
Negative	263	169	94	
Vaginal invasion				0.533
Positive	43	26	17	
Negative	237	155	82	

FIGO, the International Federation of Gynecology and Obstetrics; LVSI, lymphovascular space invasion; LNM, lymph node metastasis; BMI, body mass index. ^a^ The immunostaining scores of PRDX3

**Table 2:** Multivariate logistic regression analyses of factors associated with lymph node metastasis.

Variables^a^	HR (95% CI)	*P*
LVSI		0.004
Negative (reference)	1	
Positive	3.666 (1.518-8.851)	
Tumor size (cm)		0.001
≤ 4	1	
> 4	1.707 (1.239-2.353)	
PRDX3		0.001
Low (reference)	1	
High	3.491 (1.698-7.176)	

HR, hazard ratio; 95% CI, 95% confidence interval; FIGO, the International Federation of Gynaecology and Obstetrics; LVSI, lymphovascular space invasion. For the stepwise multivariate analysis, Forward LR method was used to select significant variables. ^a^ Variables entered for analysis were: age, tumor size, differentiation, FIGO stage, LVSI, stomal invasion, parametrial invasion, vaginal invasion, PRDX3.

**Table 3:** Sensitivity, specificity, AUC, positive and negative predictive values of factors associated with lymph node metastasis.

Factors	Sensitivity (95% CI) (%)	Specificity (95% CI) (%)	PPV (95% CI) (%)	NPV (95% CI) (%)	AUC (95% CI)
LVSI	28.57 (16.6-43.3)	90.48 (85.9-93.9)	38.9 (22.9-56.8)	85.7 (80.6-89.8)	0.595 (0.501-0.689)
Tumor size	77.55 (63.4-88.2)	51.08 (44.4-57.7)	25.2 (18.5-32.9)	91.5 (85.3-95.7)	0.678 (0.601-0.755)
PRDX3	61.22 (46.2-74.8)	70.13 (63.8-76)	30.3 (21.5-40.4)	89.5 (84.1-93.6)	0.657 (0.570-0.743)
PRDX3+LVSI+ Tumor size	91.84 (80.4-97.7)	61.47 (54.9-67.8)	33.6 (25.7-42.2)	97.3 (93.1-99.3)	0.826 (0.770-0.883)

PPV, positive predictive value; NPV, negative predictive value; AUC, the area under the receiver operating characteristic curve; 95% CI, 95% confidence interval

**Table 4:** Multivariate Cox's proportional hazards model analysis of relapse-free and overall survivals.

Variables	Overall survival		Relapse-free probability	
	HR (95% CI)	*P*	HR (95% CI)	*P*
Age (year)	1.030 (1.006-1.055)	0.014		
Tumor size (cm)	1.332 (1.118-1.587)	0.001	1.286 (1.087-1.521)	0.003
LNM		< 0.001		< 0.001
Negative (reference)	1		1	
Positive	2.997 (1.850-4.855)		2.985 (1.883-4.733)	
LVSI		0.023		0.012
Negative (reference)	1		1	
Positive	1.847 (1.089-3.132)		1.916 (1.156-3.174)	
PRDX3		0.001		<0.001
Low (reference)	1		1	
High	2.143 (1.340-3.427)		2.594 (1.632-4.122)	

HR, hazard ratio; 95% CI, 95% confidence interval; LNM, lymph node metastasis For the stepwise multivariate analysis, forward LR method was used to select significant variables. Variables entered for analysis were the following: age, FIGO stage, tumor size, LNM, BMI, LVSI, differentiation, stromal invasion, vaginal invasion, parametrial invasion, PRDX3.
