# Supplementary material for: PRDX3 Promotes Lymph Node Metastasis in Cervical Cancer by Activating NF-κB Signaling Pathway and Anoikis Resistance
Source: Int J Med Sci. 2025 Aug 22;22(15):3839–53. doi: 10.7150/ijms.118912 (PMC12492372; doi:10.7150/ijms.118912)
Supplement: Supplementary file 1 — Supplementary tables. [file ijmsv22p3839s1.pdf]

Supplementary materials

Table S1. The sequences of primers used in this study

Primers	Sequences
PRDX3-F	5'-GAAGTTGTCGCAGTCTCAGT-3'
PRDX3-R	5'-TGATCTTAGTGCAAGACCAGA-3'
GAPDH-F	5'-AGAAGGCTGGGGCTCATTTG-3'
GAPDH-R	5'-AGGGGCCATCCACAGTCTTC-3'

Table S2. The antibodies used in this study

Target protein	Antibody	Application
PRDX3	Proteintech; 10664-1-AP	WB; IHC
GAPDH	Proteintech; 10494-1-AP	WB
NF-κB p65	Abcam; ab288751	WB
Phospho- IκB-α	CST; #2859	WB
IκB-α	CST; #4814	WB
MMP-9	Proteintech; 30592-1-AP	WB
VEGF-C	Proteintech; 22601-1-AP	WB
Nuclear matrix protein p84	Abcam; ab487	WB
β-actin	Proteintech; 20536-1-AP	WB
Pan-cytokeratin	Proteintech; 26411-1-AP	IHC
LYVE-1	Abcam; ab218535	IHC
Bax	CST; #2772	WB
Bcl-2	CST; #3498	WB
Bcl-xL	CST; #2764	WB

Table S3. The siRNA sequences used in this study

siRNAs	Sequences
siPRDX3-1	sense： 5'-CCAUCUUGCCUGGAUAAAUTT-3'<br>antisense： 5'-AUUUAUCCAGGCAAGAUGGTT-3'

siPRDX3-2	sense：	5'-GGUUCUGGUCUUGCACUAATT-3'
	antisense：	5'-UUAGUGCAAGACCAGAACCTT-3'
